# Efficacy Analysis of Endovascular Therapy for Nonthrombotic Iliac Vein Compression Syndrome Combined with Chronic Venous Insufficiency

**DOI:** 10.1155/2022/2718314

**Published:** 2022-07-26

**Authors:** Renda Zhu, Xiaodong Jin, Jiayi Shen

**Affiliations:** Department of General Surgery, Haining People's Hospital, Zhejiang Province, Haining, 314400 Zhejiang, China

## Abstract

**Purpose:**

This research is aimed at elucidating the clinical efficacy of balloon dilatation (BD) plus stent implantation for nonthrombotic iliac vein compression syndrome (NIVCS) combined with chronic venous insufficiency (CVI) in different compression positions.

**Methods:**

Sixty-five NIVCS patients comorbid with CVI admitted between December 2015 and April 2020 were selected and assigned to two groups according to different iliac vein compression positions. Both groups of patients received iliac vein BD + stent implantation, with the difference lying in that the tip of the stent was inserted 0.5-1 cm into the inferior vena cava (IVC) in the experimental group versus 2-3 cm in the control group. The technical success rate, the postoperative venous clinical severity score (VCSS), and the incidence of complications were compared.

**Results:**

The technical success rate of both groups was 100%. Patients were followed up for 12-36 months (average: 25.5 ± 6.2). Decreases in VCSS were observed in both cohorts at 3, 6, 12, 24, and 36 months postoperatively compared with the preoperative scores, but with no statistical difference. There was no death, nor related complications such as restenosis and lower limb deep vein thrombosis during the follow-up period, with no statistical difference in the incidence of complications between groups.

**Conclusions:**

BD + stent implantation is a safe and effective treatment for NIVCS with few complications and remarkable short-term and medium-term effects.

## 1. Introduction

Nonthrombotic iliac venous compression syndrome (NIVCS) refers to a condition in which the left common iliac vein or inferior vena cava (IVC) is compressed by the right common iliac artery and the fifth lumbar vertebra simultaneously, causing the venous return disorder of the left lower extremity [1, 2]. It is usually manifested as chronic venous insufficiency (CVI) such as superficial varicose veins, swelling, pain, and skin ulcers of the lower extremities, especially in the left lower limb [3]. According to clinical symptoms, it can be divided into either asymptomatic, CVI, or iliofemoral venous thrombosis type, among which the first two types belong to NIVCS [4]. According to relevant epidemiological data, about 24% of patients with iliac venous compression syndrome (IVCS) are asymptomatic, and clinical symptoms may appear when the disease is severe, posing a serious threat to the physical and mental health and quality of life of such patients [5, 6]. Iliac vein balloon dilatation (BD) and stent implantation, which can correct obstructive lesions, relieve clinical symptoms, and reduce the risk of secondary deep vein thrombosis of lower limbs with a high mid- and long-term patency rate [7], have gradually become the preferred treatment for iliac vein stenosis and occlusion. In recent years, endovascular technology has become the preferred choice for NIVCS because of its advantages of minimal invasiveness, obvious clinical efficacy, few complications, and high technical success rate. At present, there is a growing consensus on endovascular treatment and primary stent implantation after BD [8–11]. But still, there are controversies about the positioning of the tip of stent implantation and the length of stent implantation into the IVC due to the difference of the iliac vein compression sites or the anatomical position of the iliac vein compression point [12], which may lead to different postoperative complications. This study retrospectively analyzed the postoperative efficacy of 65 patients undergoing endovascular treatment for NIVCS in the Department of Vascular Surgery, Haining People's Hospital, from December 2015 to April 2020, and conducted a single-center efficacy analysis for endovascular treatment of NIVCS, aiming to provide reliable clinical evidence for the optimization of treatment techniques and improve the prognosis of such patients.

## 2. Materials and Methods

### 2.1. General Information

A total of 65 patients, including 40 males and 25 females, aged 36-81 years (mean: 64.2 ± 11.4), were diagnosed with left lower limb lesions, mainly presenting with varying degrees of chronic CVI symptoms such as pain, edema, varicose veins, pigmentation, and ulcer of the affected limb. All cases underwent digital subtraction angiography (DSA), the recognized gold standard for the diagnosis of IVCS [13], because DSA can clearly display the anatomical structure of the stenotic segment of the iliac vein and the function of deep vein valves and venous reflux of lower extremities and more intuitively show the degree and causes of stenosis. Generally, the diagnosis can be made by lower extremity DSA. And femoral vein catheterization was performed if DSA failed to diagnose the disease, and the diagnosis was based on the widening of left and right diameters of the iliac vein, weak contrast agent at the compression site, reduction of anterior and posterior diameters, and establishment of pelvic collateral circulation. Patients' enrollment criteria are as follows: (1) patients with C3 grade or above CVI according to the Clinical-Etiologic-Anatomic-Pathophysiologic (CEAP) classification [14], (2) iliac vein compression by more than 50% as indicated by intraoperative venography, (3) development of collateral circulation vessels, and (4) Patients who undergo surgical treatment with the consent for operation obtained from patients themselves or their families. Exclusion criteria are as follows: (1) lower extremity deep vein thrombosis (LEDVT) or postthrombotic syndrome; (2) presence of pelvic space-occupying lesions as indicated by pelvic color Doppler ultrasound, computerized tomography (CT), or magnetic resonance imaging (MRI); (3) patients with surgical intolerance due to heart, lung, liver, and renal insufficiency; and (4) patients with allergies to contrast agents or contraindications to anticoagulation. Patients were assigned to two groups according to the way of endovascular treatment, including 39 cases (experimental group) who underwent BD + stent implantation with the tip of the stent inserted 0.5-1 cm into the IVC and 26 cases (control group) who underwent the same procedure but with the tip of the stent inserted 2-3 cm into the IVC. The experimental group and control group were not statistically different in age, gender, CEAP classification, lesion side, and preoperative venous clinical severity score (VCSS) and were therefore comparable (Table 1). Exclusion criteria are as follows: acute LEDVT, LEDVT syndrome, pelvic space-occupying lesions, active bleeding, renal failure, patients who refuse endovascular treatment, elderly patients who cannot tolerate surgery, and those with a life expectancy less than 1 year.

### 2.2. Methods

Iliac vein compression was confirmed in both groups by preoperative lower limb venography. In the supine position, ipsilateral femoral vein puncture was performed on the affected side of the femoral vein, and a 10F vascular sheath was placed. The diagnosis and lesion were further confirmed by sheath angiography, and the guide wire was introduced to open the stenosis or occlusion segment. After confirmation, the pressure difference between the two ends of the stenosis was measured to be greater than 2 mmHg (1 mmHg = 0.133 kPa). According to the stenosis degree of the iliac vein lesion and the diameter of the proximal and distal veins of the diseased segment, a P3 (Cordis Company, USA) or 8-14 mm charge balloon (Boston Scientific, USA) was selected to fully predilate the stenosis occlusion segment step by step in both groups, until the angiography showed that the stenosis of the iliac vein lesion was less than 30%, the peripheral collateral circulation was significantly reduced, and the pressure difference between the two ends of the stenosis was less than 2 mmHg. Then, primary Wallstent (Boston Scientific, USA) implantation was performed, with the diameter of the stent 1-2 mm larger than the iliac vein, the length completely covering the diseased segment, and the proximal end exceeding the diseased segment by 5-10 mm. Due to intraoperative displacement and contraction of the stent, the ejector rob of the delivery sheath was gently pushed forward every 3-4 cm during the stent release after positioning, so that the stent can be fully deployed as far as possible until it is completely released and completely attached to the iliac vein, so as to reduce the occurrence of postoperative stent displacement. It is advisable for the stent tip not to touch the opposite side wall of the IVC. After releasing the stent, radiography was performed in the anteroposterior-lateral position to confirm whether the stent was well opened, and if not, a balloon of appropriate size was selected for postexpansion. Subcutaneous injection of low molecular weight heparin was routinely administered once a day since the 1st postoperative day. Patients were required to wear elastic socks for 6 months after discharge and were given oral rivaroxaban 20 mg once a day for 3 months. The coagulation function was tested regularly.

### 2.3. Evaluation Methods

Technical success was defined as intraoperative angiography indicating smooth blood backflow to the iliac vein and IVC, good stent opening without obvious displacement, and significantly less peripheral collateral circulation than before surgery, with the pressure difference between the two ends of stenosis less than 2 mmHg. The preoperative and postoperative VCSS, postoperative complications (stent thrombosis, bilateral LEDVT, stent displacement, etc.), and deaths in two groups were recorded and compared. The VCSS score ranges from 0 to 30, which is proportional to the venous clinical severity.

### 2.4. Statistical Methods

The data was statistically analyzed by the SPSS 20.0 (SPSS, Inc., Chicago, IL, USA) statistical software. The analyses of counting data recorded as % and measurement data expressed as $\bar{x}\pm s$ were performed by the *χ*^2^ test and *t*-test, respectively, with *P* < 0.05 as the level of significance.

## 3. Results

In this study, there were 39 cases (60%) of left common iliac vein compression and 26 cases (40%) of IVC combined with left common iliac vein compression. The technical success rate of both groups was 100%, with no death, and the majority of patients experienced significant relief or disappearance of lower limb pain 1-3 days after endovascular treatment. The lower extremity pain relief rate, swelling relief rate, and ulcer healing rate in the experimental group were 90% (18/20), 100% (39/39), and 85.7% (6/7), respectively, while those in the control group were 86.7% (13/15), 100% (26/26), and 75% (3/4), respectively, with no statistical significance between groups. The success rate of endovascular surgery was 100%. The diameter of the implanted stent was 12-16 mm, mostly 14 mm (93.8%), and the length was 90 mm. Elastic stockings were used for treatment for at least 3 months after surgery. Patients all recovered well during the 12-36 (mean: 25.5 ± 6.2) months of follow-up. Follow-up ultrasound examination showed 100% patency rate of the iliac vein, no stent displacement, stent thrombosis, or bilateral LEDVT. The results of lesion length, as well as stent diameter, stent length, and patency of patients with different types of compression, are shown in Table 2. No statistical differences were found in stent diameter, stent length, and postoperative patency between groups (*P* > 0.05). Follow-up showed that the patency rates of patients with stent diameters of 12 mm, 14 mm, and 16 mm were 100% (2 cases), 100% (60 cases), and 100% (3 cases), respectively, also showing no significant difference (*P* > 0.05). The VCSS also differed insignificantly between groups at 3, 6, 12, 24, and 36 months postoperatively (*P* > 0.05) (see Table 3 for details).

## 4. Discussion

The occurrence of iliac vein compression syndrome is a chronic process with the main clinical symptoms of lower limb swelling, varicose veins, pigmentation, lower limb ulcers, etc., which can be complicated by LEDVT and postthrombotic syndrome in severe cases [7]. Current studies suggest that correcting iliac vein compression can improve the clinical presentations of patients with CVI, irrespective of the presence of venous reflux disorder [15]. However, instead of large-sample multicenter clinical analysis of iliac vein endovascular treatment in China, most of the current studies are single-center ones.

BD and stent implantation can significantly reduce lower limb pain, edema, and ulcers, while lowering the recurrence rate of venous reflux diseases. The purpose of stent implantation is to prevent venous elastic retraction and reduce the impact of the endovascular structure damaged by percutaneous endovascular angioplasty on blood flow, so as to reduce the risk of thrombosis. Regarding the indication of stent implantation in the treatment of NIVCS, Ming et al. [16] reported that this procedure was safe and effective with a high patency rate, despite the high incidence of iliac vein stenosis. However, not all patients with iliac vein stenosis need stenting, so the indications for iliac vein stenting should be strictly followed. First of all, DSA of the iliac vein of the lower limbs should be performed to widen the left and right diameters, weaken the contrast agent at the compression site, reduce the front and back diameters, and establish the pelvic collateral circulation, with the pressure difference between the two ends of the stenosis greater than 2 mmHg at rest. At the same time, the clinical symptoms of lower limbs, mainly including CEAP grading of CVI > grade 3, obvious swelling, pigmentation, or ulcers, should be taken into consideration. Most iliac vein lesions can be diagnosed by intravascular ultrasound [17] with a positive rate significantly higher than that of angiography. The area measurement provided by intravascular ultrasound is of great significance for diagnosing iliac vein occlusion and guiding stent placement, but this procedure has not been carried out in most primary hospitals in China at present. The second is the choice of stents. Currently, Wallstents, which are the only stents that can be implanted into the venous system and the exact one we used in our center, are used in most vascular surgeries in China. The third is the positioning of the stent. Raju et al. [18] and Professor Lu Xingwu from Shanghai Ninth People's Hospital believe that the stent should be inserted into the IVC for 3-5 cm to prevent the proximal end of the stent from collapsing and stent displacement. A research found that the implantation of an excessively long stent in the IVC may affect the contralateral iliac vein blood backflow and increase the incidence of contralateral LEDVT [19], although the actual clinical incidence is not high. In the actual clinical work, intraoperative angiography found that there was compression of both the IVC and the left common iliac vein in some patients, and a simple stent entering the IVC by 10 mm could not completely cover the stenosis segment, or the tip just covered the lesion segment. The total length of a Wallstent into the IVC may be 20-30 mm because it risks stent retraction. Some vascular surgeons perform simultaneous angiography of bilateral iliac veins before stent implantation and then released the stent under the roadmap, which is more conducive to the precise positioning of the stent. We believe that this method is more effective and feasible and can be popularized in primary hospitals.

Our center summarized the reasons for the failure of stent placement in other central iliac vein occlusions in the past. In order to prevent the potential risk of too much stent implantation into the IVC that affects the contralateral blood, some surgeons did not cover venous lesions before the primary stent implantation or made the tip of the stent exceeds the lesion too little, resulting in stent tip retraction, surgical failure, and even stent thrombosis and deep venous thrombosis of the affected lower extremity. Therefore, according to our experience, the tip of a Wallstent generally needs to exceed the proximal end of the compression point by 10 mm, as there is a risk of intraoperative and postoperative displacement because of its weak radial support but good flexibility. The diameter of the selected stent is generally 12-16 mm, and the length is mostly 90 mm. Intraoperative balloon is required to fully preexpand the stenosis or occlusion segment. On the basis of completely covering the diseased segment, a 90 mm long stent is preferred to reduce the possibility of displacement. During the release process, the joystick should be gently pushed forward every 3-4 cm of release to make the mesh of the stent open as completely as possible, so that the stent can fully adhere to the iliac vein and reduce intraoperative and postoperative stent displacement. In this way, our center had significantly reduced intraoperative and postoperative stent displacement. This study determined no significant difference in stent length between different compression types. Therefore, different compression types do not seem to affect the short- and medium-term patency rate of stents, provided that the stent diameter is matched to fully cover the lesion, but follow-up reports on long-term outcomes are still lacking. The fourth issue is the diameter of the stent. It has been reported in the literature that the stent diameter has no significant impact on the patency rate and the relief of patients' symptoms [20]. The results of this study also confirm similar short- and medium-term patency rates of stents with different diameters. The size of the iliac vein stent should be avoided to be too small to cause residual symptoms. Generally, a dilatation balloon of the largest diameter corresponding to the diameter of the stent is selected in our center. The fifth is the patency of the stent. The iliac vein is prone to thrombosis due to its low pressure and slow flow rate. However, iliac vein stents have a high long-term patency rate, and the stent thrombosis rate is less than 5% for nonthrombotic iliac vein stenosis [21].

All the iliac vein stent thrombosis in our hospital were postsurgical patients with postthrombotic syndrome. None of the 65 patients included in this study had serious complications such as death, venous thromboembolism, severe bleeding at the puncture site, vascular rupture during dilatation, or operation-related infection. During the 12-36 months of follow-up, most of the venous functions of the lower limbs were significantly improved, with no serious complications (stent thrombosis, contralateral iliac femoral vein thrombosis, etc.). Therefore, it is considered that it is safe for stents to enter 3 cm of the IVC on the basis of completely covering the diseased segment. Limited by the characteristics of retrospective study, the sample size of this study was small and only supported by data from a single center, resulting in data collection bias. Besides, the habits of different surgeons may also affect the stent implantation and patency rate.

The innovations of this study are as follows: first, it confirms the efficacy, reliability, and safety of iliac vein BD plus stent implantation in the treatment of NIVCS patients with CVI from the aspects of technical success rate, VCSS scale, and safety. Second, related parameters and surgical details of this treatment technique were optimized by summarizing the reasons for previous failures of stent implantation of other central iliac vein occlusions, providing optimization means and clinical reference for the management of NIVCS patients with CVI.

## 5. Conclusion

In a word, BD combined with stent implantation is a safe and effective treatment for NIVCS with few complications and remarkable short- and medium-term effects. But there are still some controversies about the positioning before implantation and the length of the stent inserted into the IVC. We believe that full coverage of the diseased segment of the ipsilateral iliac vein and avoiding excessive length of stent insertion into the IVC should be considered at the same time during stent implantation. Large-sample studies are still required to further determine the patency rate of stents in the long term, as well as the effects of stents on complications associated with contralateral blood flow.

## Figures and Tables

**Table 1:** Comparison of general data between two groups of patients.

General data	Control group (*n* = 26)	Experimental group (*n* = 39)	*P* value
Males (*n* (%))	18 (69.23)	22 (56.41)	>0.05
Age (*x* ± *s*, years old)	65.2 ± 8.4	63.7 ± 7.2	>0.05
Lesion side (*n* (%))			
Left side	26 (100)	39 (100)	>0.05
CEAP classification (*n*)			>0.05
C3	5 (19.23)	8 (20.51)	
C4	8 (30.77)	13 (33.33)	
C5	9 (34.62)	11 (28.21)	
C6	4 (15.38)	7 (17.95)	
Preoperative VCSS ($\bar{x}\pm s$, points)	14.3 ± 2.28	14.54 ± 2.76	

**Table 2:** Comparison of related results of patients with different types of compression.

Results	Left common iliac vein compression	Left common iliac and inferior vena cava compression
Lesion length (cm)	2.1 ± 0.62	3 ± 0.41
Stent diameter (mm)	14.15 ± 0.45	14.31 ± 0.62
Stent length	90	90
Patency degree	39 (100)	26 (100)

**Table 3:** Comparison of postoperative follow-up VCSS between two groups ($\bar{x}\pm s$), points.

Group	Follow-up time				
	3 months	6 months	12 months	24 months	36 months
Experimental group	6.41 ± 1.47^∗^	5.32 ± 1.02^∗^	4.23 ± 1.15^∗^	3.57 ± 1.53^∗^	2.35 ± 1.30^∗^
Control group	6.53 ± 2.13^∗^	5.67 ± 1.34^∗^	4.37 ± 1.60^∗^	3.69 ± 1.23^∗^	2.24 ± 1.24^∗^
*P* value	>0.05	>0.05	>0.05	>0.05	>0.05

^∗^Compared with the preoperative value, *P* < 0.05.

## Data Availability

The labeled dataset used to support the findings of this study are available from the corresponding author upon request.
